# A narrative review of the pathophysiology of sepsis in sub-Saharan Africa: Exploring the potential for corticosteroid therapy

**DOI:** 10.1371/journal.pgph.0004429

**Published:** 2025-04-09

**Authors:** Phoebe Gruccio, William S. Girard, Amelia D. Badipour, Reagan Kakande, Victor Adejayan, Muhammad Zulfiqar, Michael Ndyomugabe, Philemon Ojuman, Scott K. Heysell, Megan Null, Jeffrey Sturek, Tania Thomas, Stellah Mpagama, Conrad Muzoora, Eva Otoupalova, Edwin Nuwagira, Christopher C. Moore

**Affiliations:** 1 Division of Infectious Diseases, Department of Medicine, University of Virginia, Charlottesville, Virginia, United States of America; 2 Department of Medicine, Mbarara University of Science and Technology, Mbarara, Uganda; 3 Division of Pulmonology and Critical Care Medicine, Department of Medicine, University of Virginia, Charlottesville, Virginia, United States of America; 4 Department of Medicine, Kibong’oto Infectious Diseases Hospital, Sanya Juu, United Republic of Tanzania,; 5 Division of Pulmonology and Critical Care Medicine, Department of Medicine, Tulane University School of Medicine, New Orleans, Louisiana, United States of America; 6 Tuberculosis Treatment Unit, Mbarara Regional Referral Hospital, Mbarara, Uganda; Aga Khan University, PAKISTAN

## Abstract

Sepsis remains a significant global health threat with a disproportionate burden in low-income countries including those in sub-Saharan Africa where case fatality rates are as high as 30% to 50%. Defined as a severe systemic response to infection, sepsis leads to widespread immune dysregulation and organ dysfunction, including adrenal insufficiency. Critical illness-related corticosteroid insufficiency (CIRCI) arises from dysregulation of the hypothalamic-pituitary-adrenal (HPA) axis, altered cortisol metabolism, and tissue resistance to glucocorticoids, all of which can occur during sepsis. Clinical trials of corticosteroids for the treatment of patients with sepsis and septic shock have shown improvements in shock reversal, and in some studies, patient survival; however, their role in the treatment of sepsis in sub-Saharan Africa is unknown. The incidence of sepsis in sub-Saharan Africa is compounded by high rates of human immunodeficiency virus (HIV) and co-infections, including tuberculosis (TB), which is the leading cause of sepsis. Both HIV and TB can cause immune dysregulation and adrenal insufficiency, which may exacerbate CIRCI and prolong shock. Existing sepsis research has been predominantly conducted in high-income countries and has largely excluded people living with HIV or TB. Therefore, there is a need to better understand sepsis and CIRCI pathophysiology in the context of specific regional host and pathogen characteristics. In this narrative review, we explored the pathophysiology of sepsis in sub-Saharan Africa including the existing literature on the immune response to sepsis and the prevalence of adrenal insufficiency in patients with HIV and TB, with a focus on the implications for corticosteroid management. We found a compelling need to further evaluate corticosteroids for the treatment of sepsis in Africa.

## Introduction

Sepsis is the leading cause of global death, and affects an estimated 48.9 million people annually [1]. Sepsis is defined as life-threatening organ dysfunction caused by a dysregulated host response to infection, and can lead to septic shock, during which underlying circulatory, cellular, and metabolic abnormalities cause end-organ damage and death. Sepsis can be caused by a wide range of infections, which can be precipitated or exacerbated by injuries or non-communicable diseases [2–4]. The immune response to sepsis includes both hyperinflammatory and immunosuppressive subphenotypes [5]. Understanding host-pathogen interactions is critical for developing effective interventions and improving patient outcomes [5].

The largest global sepsis burden is borne by low- and middle-income countries (LMICs). In 2017, approximately 85% of sepsis cases and 87% of deaths due to sepsis occurred in LMICs [1]. A high incidence of infection combined with resource-constrained public health efforts and low vaccination rates, undernutrition, and limited human and material resources contribute to the disproportionately high morbidity and mortality from sepsis in LMICs [2]. In sub-Saharan Africa, the burden of sepsis can be attributed in part to the high prevalence of human immunodeficiency virus (HIV) and co-infections with *Mycobacterium tuberculosis* (Mtb), non-Typhoid *Salmonella*, and *Streptococcus pneumoniae* [6]. For example, one Ugandan study found that 85% of patients admitted with sepsis were living with HIV [7], while Mtb is estimated to cause approximately 30% of sepsis in sub-Saharan Africa [8].

Adrenal insufficiency (AI) is caused by the inability of the adrenal cortex to produce sufficient amounts of glucocorticoids or mineralocorticoids. A diagnosis of AI is based on the presence of low early-morning serum cortisol or inappropriate stimulated serum cortisol concentration in the presence of elevated adrenocorticotropic hormone (ACTH) concentrations [9]. Many cases of AI go undiagnosed due to its nonspecific and often subtle signs and symptoms such as fatigue, weight loss, abdominal pain, nausea, vomiting, and myalgias, which often overlap with those of HIV or tuberculosis (TB) [10]. Critical illness related corticosteroid insufficiency (CIRCI) is more broadly defined as dysfunction of the hypothalamic-pituitary-adrenal axis that occurs in 10% to 20% of critically ill medical patients and in as high as 60% of patients with septic shock [5,11]. CIRCI occurs as a result of AI or tissue resistance to glucocorticoids and is characterized by inadequate cellular corticosteroid activity and an exaggerated proinflammatory response for the severity of the patient’s illness [5,11,12].

In sub-Saharan Africa, HIV, TB, and cytomegalovirus (CMV) reactivation are commonly found in patients with sepsis, all of which can contribute to AI [13]. Sub-Saharan Africa has the highest prevalence of people living with HIV (PLWH), and has the second highest global TB incidence [14]. Approximately 32% of people with TB are also PLWH [15]. As we seek to enhance sepsis management and optimize patient outcomes in specific patient populations, we must understand their pathophysiological underpinnings and their burden of sepsis. Despite the high burden of sepsis in sub-Saharan Africa, there are limited published clinical data from Africa on the pathophysiology of sepsis or the use of corticosteroids in sepsis. Given this knowledge gap, we aimed to describe the immunopathophysiology of sepsis, CIRCI, and AI in the context of host and pathogen characteristics, and the potential role for corticosteroids in the treatment of sepsis and septic shock in sub-Saharan Africa.

## Methods

**Fig 1:**
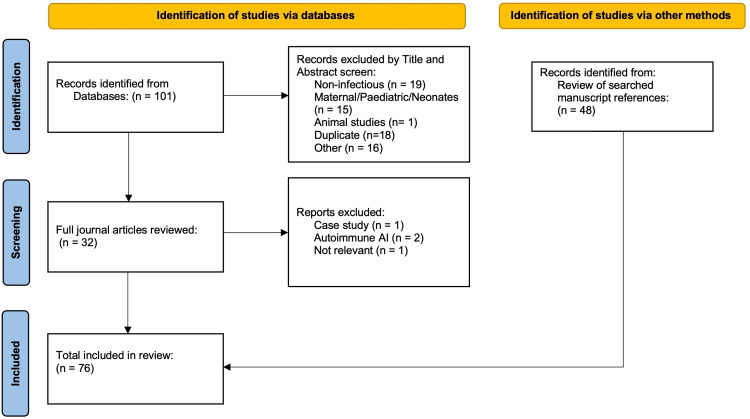
Flowchart of the literature review of journal articles that focused on sepsis immunopathophysiology, or adrenal insufficiency or corticosteroid use in critical illness within sub-Saharan Africa, 2000-2024 in accordance with the PRISMA 2020 flow diagram for new systematic reviews.

On April 10, 2024, we searched PubMed, CINAHL, Ovid MEDLINE, Scopus, and African Journals Online for journal articles on steroid management and adrenal insufficiency in critical illness conducted in sub-Saharan Africa from January 2000 to July 2024. Our results were from a keyword search of the topics of “adrenal insufficiency,” “adrenalitis,” “hypoadrenalism,” “immune phenotypes,” “human immunodeficiency virus or HIV,” “tuberculosis or TB,” “cytomegalovirus or CMV,” “sepsis,” “septic shock,” “critical illness,” “steroids,” “corticosteroids,” plus “sub-Saharan Africa” or “Africa south of the Sahara.” We defined sub-Saharan Africa based on the World Bank regional classification, which includes countries in the African continent south of the Sahara [16]. In addition to the database search, we reviewed references from relevant journal articles. A flow chart of included studies in accordance with PRISMA 2020 guidelines is provided in Fig 1 [17]. We provided an example of the search strategy in S1 Table and a comprehensive list of the 57 studies included in the narrative review in S2 Table.

Two authors (P.G. and W.S.G.) performed the study selection procedure, including the preliminary search, title and abstract screening, and full-text screening. After removing duplication, they screened titles and abstracts after considering the inclusion and exclusion criteria of the studies. P.G. and W.G. assessed the full-text articles, and C.C.M. resolved any disagreements regarding inclusion of articles.

## Results

### Current sepsis management in sub-Saharan Africa

The Surviving Sepsis Campaign, established in 2004 [18], and most recently updated in 2021 [19], has raised awareness about the importance of early intervention in sepsis management. The Surviving Sepsis guidelines are based on trials conducted in HICs, and are therefore often difficult to implement in LMICs due to their demanding requirements for material and human resources. Additionally, sepsis trials conducted in HICs have not frequently included patients with HIV or TB, which limits their applicability to sub-Saharan Africa [2]. Recognizing these disparities, a panel convened at the 23rd European Society of Intensive Care Medicine (ESICM) congress in 2010 developed modified recommendations tailored for resource-limited environments. The panel adjusted definitions for severe sepsis and septic shock to better align with commonly available diagnostic resources. The panel similarly emphasized the need for immediate intervention in the treatment of sepsis. However, uncertainty regarding optimal diagnosis and management of sepsis in sub-Saharan Africa remains [2].

Early identification and risk-stratification of sepsis remains a challenge in low-resource settings including sub-Saharan Africa [2]. The modified early warning score (MEWS), the quick Sequential (Sepsis-Related) Organ Failure Assessment (qSOFA) score and the Universal Vital Assessment (UVA) score are bedside tools to predict sepsis mortality [20]. Unlike MEWS and qSOFA, the UVA score was intentionally derived and validated to predict in-patient mortality in sub-Saharan Africa using easily obtainable clinical data from hospitalized patients in six African countries [21]. The UVA score has been extensively externally validated in Africa and in parts of Asia where it has outperformed qSOFA and MEWS [21–24]. Understanding the severity of patient illness by initial calculation followed by serial monitoring of the UVA score may help better identify the most severely ill patients with sepsis who would benefit from adjunctive corticosteroid therapy [20,25].

PLWH with sepsis are at particularly high risk of mortality, yet the optimal management of sepsis in this vulnerable population is unclear. A 2023 systematic review found limited evidence to inform the diagnosis and management of hospitalized PLWH in low-resource settings [26]. Given the high prevalence of TB in these patients and the importance of early treatment, the review suggested that urine lipoarabinomannan assay (TB-LAM), a cell wall biomarker associated with disseminated TB, and other rapid TB screening tests such as the nucleic acid based Mtb/RIF Gene Xpert test may reduce mortality of hospitalized PLWH [27,28]. Fluid resuscitation is a key component of sepsis management recommendations from HICs. However, two randomized clinical trials of fluid resuscitation in sub-Saharan Africa showed increased mortality with protocolized fluid resuscitation in adults and children with septic shock who received fluid or albumin boluses, respectively [29,30]. These findings suggest that even widely established HIC interventions, such as initial fluid resuscitation in sepsis, may be harmful in settings with limited intensive care unit and mechanical ventilator availability.

The 2016 and 2021 Surviving Sepsis guidelines recommended treating septic shock in patients who are unable to achieve hemodynamic stability with fluids and vasopressors with corticosteroids [19,31]. In 2019, Mer et al. reviewed and adapted sepsis management recommendations from high-resource settings for LMICs, and included the use of low-dose hydrocortisone (200 mg) for adults with refractory shock [32]. Ease of use, low cost, and availability make corticosteroids an attractive option for the treatment of septic shock in resource-limited settings; however, no clinical trial has tested their use in sepsis in sub-Saharan Africa and their use has not been widely implemented [32]. Potential risks, including immunosuppression and increased susceptibility to co-infections, may complicate their effectiveness in sepsis management in Africa. These challenges emphasize the need for clinical trials to better understand the role of corticosteroids in sepsis treatment in sub-Saharan Africa.

### The immune response to sepsis

**Fig 2:**
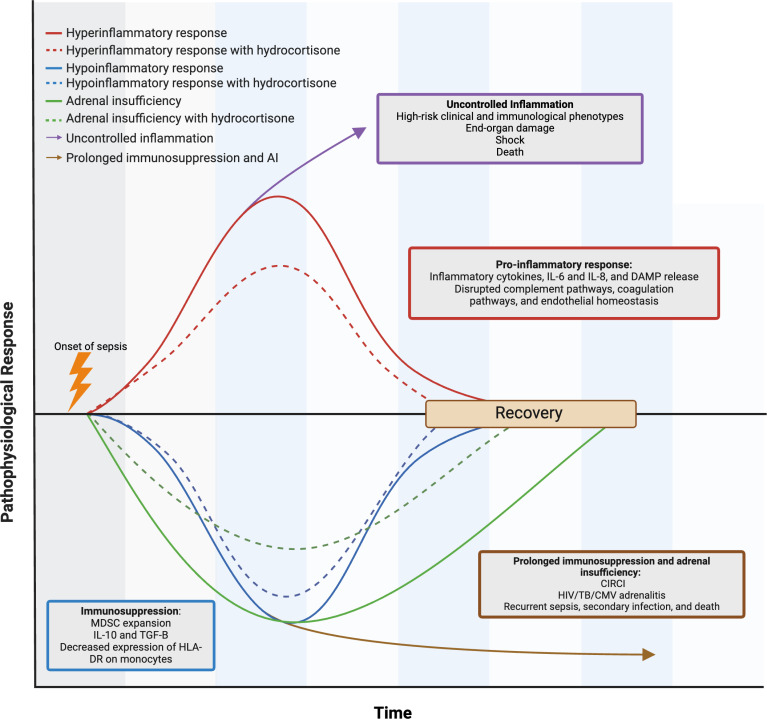
The expected effect of hydrocortisone on sepsis pathophysiology in patients in sub-Saharan Africa. The figure illustrates how hydrocortisone can regulate the immune dysfunction seen in sepsis, which is marked by both hyper- and hypo-inflammatory responses. Hydrocortisone mitigates the hyperinflammatory response by reducing the release of excessive proinflammatory cytokines and enhancing immune function, which can lead to a decreased compensatory hypo-inflammatory response. In sub-Saharan Africa, high-risk clinical and immunological phenotypes contribute to poor sepsis outcomes, primarily due to uncontrolled hyperinflammation in the setting of HIV and TB. The figure also depicts hydrocortisone’s role in ameliorating critical illness-related corticosteroid insufficiency that is worsened by frequently co-prevalent HIV, TB, and CMV adrenalitis in sub-Saharan Africa. CIRCI, critical illness-related corticosteroid insufficiency; CMV, cytomegalovirus; DAMP, damage-associated molecular patterns; HLA-DR, human leukocyte antigen-differentiation region; IL, interleukin; MDSC, myeloid-derived suppressor cell; TGF-β, transforming growth factor beta.

Sepsis spurs concurrent proinflammatory and anti-inflammatory immune responses, where persistent hyperinflammation and prolonged immune hyporesponsiveness lead to cell death, organ failure, and patient mortality [33]. Inflammation in sepsis is initiated by pathogen- and damage- associated molecular patterns (PAMPs; DAMPs), which are detected by pattern recognition receptors. This activation triggers an inflammatory cascade involving cytokines, neuroendocrine system modulation, complement, coagulation systems, and lipid mediators. These processes disrupt coagulation pathways and endothelial homeostasis, leading to systemic vasodilation, increased capillary permeability, and tissue ischemia, ultimately resulting in organ dysfunction, worsening shock, and patient death [5]. Sepsis also stimulates compensatory anti-inflammatory responses, which if prolonged, can result in a chronic syndrome characterized by protein catabolism, increased cell death, late opportunistic hospital infections, and reactivation of latent viruses including CMV. In a regulated immune response, mechanisms that promote healing and minimize tissue damage work in harmony to restore immune balance. As depicted in Fig 2, this balance is disrupted during sepsis; however, immunomodulators may restore immune balance and hasten clinical recovery when inflammatory and anti-inflammatory processes do not conclude after the microbial threat has been addressed.

### Subphenotypes of the immune response to sepsis

The use of muti-omic technologies has sparked increased interest in finding biomarkers that can identify specific sepsis subphenotypes, potentially leading to more personalized treatments. C-reactive protein and other acute phase reactants are examples of more widely available biomarkers. C-reactive protein elevation was associated with greater benefits from glucocorticoids in two randomized trials of corticosteroids for pneumonia [34,35]. Several studies associated a hyperinflammatory sepsis subphenotype with the presence of elevated serum concentrations of pro-inflammatory cytokines including interleukin (IL)-6 and IL-8 [36–38], and consequently elevated IL-6 and IL-8 concentrations identified patients at higher risk of mortality [39,40]. Similarly, elevated concentrations of serum IL-10, and decreased expression of TGF-β and cell surface receptor HLA-DR on monocytes can potentially identify an immunosuppressed sepsis subphenotype with higher mortality risk [41,42]. Immune subphenotyping using a single marker remains imprecise due to a lack of standardization and varying metrics across different studies [5].

Transcriptomic signatures are an emerging tool for sepsis immuno-subphenotyping. A study of gene expression in peripheral blood leukocytes from adults with sepsis identified distinct sepsis response transcriptomic signatures (SRS1 and SRS2). SRS1, which was associated with an immunosuppressed subphenotype, demonstrated features such as endotoxin tolerance and T-cell exhaustion, and was linked to higher mortality compared to SRS2 [43]. Another approach is deep clinical subphenotyping of sepsis. Machine learning allows previously unprecedented analysis of clinical sepsis subphenotypes. In a large cohort study of more than 20,000 patients, four distinct clinical subphenotypes were identified, each showing significant variations in available sepsis biomarker patterns and case fatality rates. The most prevalent subphenotype (α) was associated with the lowest vasopressor use and had a 28-day case fatality rate of 5%. In contrast, the subphenotype with the highest mortality (δ) exhibited more liver dysfunction and septic shock, with a 28-day case fatality rate of 40% [44]. These findings suggest that recognizing and classifying sepsis subphenotypes could enhance patient management and inform clinical trial designs.

### Subphenotypes of the immune response to sepsis in sub-Saharan Africa

In LMICs, the burden of sepsis is compounded by unique host-pathogen profiles, including HIV, Mtb, malaria, and cryptococcal infections. Factors including age, infectious organism, site of infection, host genetics, treatments, and illness dynamics further alter the immune response and may influence sepsis outcomes. Therefore, there is a critical need for research in sub-Saharan Africa to identify host-response subphenotypes, particularly in relation to HIV and TB [45,46].

In several studies performed to characterize unique regional sepsis subphenotypes in sub-Saharan Africa, the presence of HIV and Mtb co-infection was found to be associated with a subphenotype characterized by increased inflammation and higher mortality [47–49]. In an early Ugandan study of sepsis and urine TB-LAM, there was an association between higher grades of positive TB-LAM results and a proinflammatory host immune response, as well as greater organ dysfunction and shock [48]. These results suggest that a higher burden of Mtb infection is associated with more severe illness. Patients with higher grades of TB-LAM exhibited heightened innate and T-cell activation, and had reduced expression of genes associated with antibacterial defense. In a subsequent cohort study from a public referral hospital in Uganda, previously described transcriptomic signatures SR-1 and SR-2 were used to distinguish different sepsis subphenotypes [43,49]. The proinflammatory signature SR-1 included mostly PLWH and was associated with increased mortality. Despite considerable differences in host-pathogen profiles in sub-Saharan Africa, this study indicated that endotypes found in LMICs shared some biological and clinical features with those seen in HICs [49].

A study from Ghana similarly investigated the transcriptomal response to sepsis [50]. The study tracked gene expression over time to determine how the host’s immune response was associated with mortality. Principal component analysis identified distinct transcriptional patterns associated with sepsis progression and recovery. Neutrophilia, lymphopenia, and CD14+ monocytes were associated with impaired cellular immunity and increased mortality. Like the previously described Ugandan cohort, the Ghanaian cohort was most aligned with the high-mortality SRS1 group at enrollment, with survival being associated with a decreasing likelihood of being in the SRS1 group over time.

Several other high-risk sepsis subphenotypes have been identified in sub-Saharan Africa. In a Ugandan study, patients with HIV were more likely to exhibit a high-risk subphenotype associated with myeloid-derived suppressor cell expansion and immunosuppression [46]. Another study conducted in Uganda identified soluble tumor necrosis factor receptor 1 (sTNFR1) and angiopoietin 2 (Ang-2) as key predictors of mortality, and when these markers were combined with the clinical UVA mortality risk score, mortality prediction improved. High-risk patients identified by the UVA score were characterized by T-cell exhaustion and upregulation of proinflammatory innate immune pathways, including Toll-like receptors and nuclear factor kappa-light-chain-enhancer of activated B cells (NFκB) [47].

### Pathophysiology of adrenal insufficiency and critical illness-related corticosteroid insufficiency (CIRCI)

While autoimmunity is the leading cause of AI in northern Africa and Caucasians in South Africa, HIV, and TB predominate as the etiologies of AI in sub-Saharan Africa. However, the true prevalence of AI in sub-Saharan Africa remains unknown, partly due to difficulties in accessing diagnostic tests and the masking of symptoms by often coexisting infections [13]. Symptoms of AI may include fatigue, weakness, lethargy, fever, abdominal pain, nausea, vomiting, and diarrhea, which are also often present with HIV and TB [13,51]. Furthermore, hyperpigmentation of the skin indicative of AI may be challenging to recognize in patients with darker skin tones [13,51].

A 2008 international task force defined CIRCI as a usually reversible condition caused by proinflammatory mediators; however, it may also arise due to structural damage of the adrenal gland. CIRCI may disrupt the balance between proinflammatory and anti-inflammatory pathways and thereby exacerbate immune, metabolic, vascular, and organ dysfunction [12]. Dysregulation of cortisol production and the corticotropin-releasing factor (CRH)/ACTH pathway is initiated by a rapid increase in ACTH levels in response to critical illness and is amplified by the complex interplay among the CRH/ACTH pathways, autonomic nervous system, and immune systems [12]. In addition to primary AI, reduced plasma clearance and tissue resistance to cortisol are additional CIRCI etiologies and may complicate diagnosis in the setting of normal plasma cortisol.

The 2017 ESICM taskforce suggested that a change in baseline cortisol at 60 min of <9 μg/dL after ACTH (250 μg) administration or a random plasma cortisol of <10 μg/dL can be used to diagnose CIRCI [52]. Among the studies included in our review, the diagnostic testing for AI was inconsistent and AI was variably defined by a subnormal response to a 1 μg or 250 µg ACTH stimulation test, or low serum cortisol levels without ACTH stimulation.

### Prevalence of adrenal insufficiency in patients living with HIV in sub-Saharan Africa

AI in PLWH often results from direct damage to the adrenal glands by HIV. Common causes of AI in this patient population include opportunistic infections such as Mtb, CMV, *Pneumocystis jirovecii* pneumonia, toxoplasmosis, and cryptococcosis, as well as neoplasms including lymphomas and Kaposi’s sarcoma. Among patients with HIV, CMV adrenalitis is the most common infectious cause of AI following Mtb [53,54]. We identified 5 studies on the prevalence of AI in PLWH from sub-Saharan Africa that took place from 2007-2018. The prevalence of AI in studies that used the 1 μg ACTH stimulation test ranged from 5-35% [55–57]. In studies using serum cortisol levels without ACTH stimulation, the prevalence of AI ranged from 19-31% [57–59]. None of the studies used the 250 µg ACTH stimulation test. One group reported a prevalence of AI of 35% among PLWH in 2013 but only 5% among PLWH and TB in 2017 despite similar methodology [55,56]. We attributed the variability in the prevalence of AI among PLWH to differences in the definition of AI or hypoadrenalism, diagnostic methodologies, variation in the severity of illness, and the challenges associated with recognizing AI due to its nonspecific symptoms [55–60]. A summary of the studies in PLWH included in our review is provided in Table 1.

**Table 1:** Summary of data on the prevalence of adrenal insufficiency (AI) associated with HIV in sub-Saharan Africa from 2007-2018.

Study	Country	Enrolled patients	Criteria for AI	Prevalence of AI	Additional relevant findings
**Meya (2007)** [59]	Uganda	113 hospitalized patients with HIV	Morning total serum cortisol level ≤ 25 µg/dl	19%	The major admitting diagnosis among both groups was tuberculosis (38% in patients with AI and 41% in those without). Risk factors for AI included rifampicin, septrin, tachycardia, HIV stage IV disease, eosinophilia, and hyponatremia. Additional major admitting diagnoses of the AI patients included Kaposi sarcoma, cryptococcal meningitis, bronchopneumonia, and *Pneumocystis jiroveci*.
**Ekpebegh (2011)** [58]	South Africa	66 hospitalized patients with HIV	Basal cortisol level < 400 nmol/L	27%	All participants (100%) were co-infected with CMV, and 68% were also co-infected with tuberculosis.
**Odeniyi (2013)** [56]	Ghana	43 recently diagnosed HIV patients	1 μg ACTH stimulation test stimulated serum cortisol < 380 nmol/L and increment from basal to stimulated cortisol level <158.5 nmol/L	35%	If a higher threshold of 500 nmol/L for the ACTH stimulation test was used, 89% of the participants would be classified as having suboptimal adrenal function. The study opted for more conservative criteria to avoid overestimating AI in this population.
**Odeniyi (2017)**[55]	Ghana	40 Patients co-infected with HIV and TB	1 μg ACTH stimulation test 30 min cortisol level <380.2 nmol/L and increment from basal to stimulated cortisol level <158.5 nmol/L	5%	The study did not observe differences in basal cortisol levels between TB patients and healthy individuals and suggested that the ACTH stimulation test may be a more sensitive measure for detecting adrenal insufficiency in this context.
**Akase (2018)** [51]	Nigeria	350 adult patients recruited from an HIV clinic	Basal cortisol level <100 μg/L 1 μg ACTH stimulation test stimulated serum cortisol <180 μg/L	31% 16%	The study found no difference in clinical features between groups. There was no correlation between CD4 count or WHO clinical stage and hypoadrenalism.

### Prevalence of adrenal insufficiency in patients with tuberculosis in sub-Saharan Africa

AI due to TB develops as pulmonary TB progresses to extrapulmonary TB, which can damage the adrenal glands and increase proinflammatory cytokines that disrupt cortisol synthesis. In addition, rifampicin, a commonly used anti-TB drug, is a potent cytochrome P450 enzyme inducer, which accelerates cortisol metabolism in the liver, thereby exacerbating cortisol insufficiency [60,61]. A comprehensive systematic review that included data from studies conducted over 31 years across sub-Saharan Africa found a prevalence of subclinical AI in patients with pulmonary TB that ranged from 1 to 60% [62]. We identified 8 studies of AI in TB from sub-Saharan Africa that were conducted from 2000-2020. The prevalence of AI among the studies ranged from 4-60%, which we attributed to differences in the definition of AI or hypoadrenalism, diagnostic methodologies, variation in the severity of illness, and the challenges associated with recognizing AI due to its nonspecific symptoms [55,63–70]. The prevalence of AI among the studies that used the 1 μg ACTH stimulation test, the 250 µg ACTH stimulation test, or serum cortisol levels without ACTH stimulation ranged from 5-23%, 4-40%, and 35-60%, respectively [55,63–70]. Although the studies that used serum cortisol levels without ACTH stimulation reported the highest prevalence of AI, several other studies found that basal cortisol levels among patients with TB were within normal range, even in patients with a subnormal response to ACTH stimulation [55,68]. A summary of each of the studies in people with TB identified in our review is provided in Table 2 [55,63–70].

**Table 2:** Prevalence of data on the prevalence of adrenal insufficiency (AI) associated with tuberculosis in sub-Saharan Africa from 2000-2020.

Study	Country	Enrolled participants	Criteria for AI	Prevalence of AI	Additional relevant findings
Kaplan (2000) [70]	South Africa	40 patients hospitalized with pulmonary TB (55% co-infected with HIV)	1 μg ACTH stimulation test stimulated serum cortisol of <414 nmol/l	5%	Argued that the 250 μg ACTH stimulation test lacks sensitivity for diagnosis of hypoadrenalism.
Venter (2006) [67]	South Africa	20 patients hospitalized with TB	Intravenous 250 µg ACTH stimulation test stimulated serum cortisol of <250 nmol/l	40%	The study concluded that rifampicin did not additionally contribute to AI in the first 5 days of treatment
Beadsworth (2008) [66]	Malawi	51 patients hospitalized with TB	Intramuscular 250 µg ACTH stimulation test stimulated serum cortisol <550 nmol/l at 30 minutes	4%	
Broodryk (2010) [65]	South Africa	73 patients with pulmonary TB	Intramuscular 250 µg ACTH stimulation test stimulated serum cortisol <500 nmol/L	7%	
Odeniyi (2011) [68]	Nigeria	70 patients with sputum smear-positive TB	1 μg ACTH stimulation test compared to healthy control group	23%	Same study as from Table 1, co-infected HIV cohort only.
Namulema (2013) [69]	Uganda	200 patients admitted with pulmonary TB	1 μg ACTH stimulation test stimulated serum cortisol level <18µg/dL	2%	
Odeniyi (2017) [55]	Nigeria	44 patients with pulmonary TB and 40 patients with pulmonary TB and HIV coinfection	1 μg ACTH stimulation test 30 min cortisol level <380.2 nmol/L and increment from basal to stimulated cortisol level <158.5 nmol/L	19%	32% of the participants with pulmonary TB had subnormal adrenal response; however, 5% of coinfected HIV patients had a subnormal response. Basal cortisol levels were normal among both groups.
Naggirinya (2020) [63]	Uganda	272 patients hospitalized with TB (57% co-infected with HIV)	Early morning serum cortisol level < 414 nmol/L	60%	Reported the highest incidence of adrenal insufficiency of the included studies. Lower basal cortisol levels were associated with drug resistant TB
Mabuza (2020) [64]	South Africa	75 patients hospitalized with suspected TB	Serum cortisol level < 500 nmol/L	35%	56% of the AI group tested positive for TB versus 44% in the adrenal sufficient group. The study reported a negative correlation between CD4+ T-cell concentration and serum cortisol.

### Role of corticosteroids in sepsis management

In the context of septic shock, corticosteroids have pleiotropic effects including improving vascular reactivity to alpha agonists, immunomodulatory effects including decreased production of pro-inflammatory cytokines and modulators, and effects on sodium and water retention [71]. On the cellular level, glucocorticoids activate genes associated with chemokines, cytokines, complement, repress genes associated with adaptive immunity, and activate or repress certain T-helper subsets. Glucocorticoids also repress NFκB, a key pro-inflammatory mediator [72,73], and decrease plasma levels of IL-6 and IL-8. Given that there are numerous redundant inflammatory pathways in the host response to sepsis and most immunomodulation therapies target only a single pathway, there may be limits to their efficacy and overall net effect on clinical outcomes [4]. However, broadly acting immunomodulators, such as corticosteroids, may be beneficial in restoring homeostasis during specific phases of sepsis response and in subgroups of patients with hyperinflammatory phenotypes. Different timing and variability in host-response subphenotypes may explain some of the variability in clinical trials of corticosteroids in sepsis [74–78].

The results of trials of corticosteroid use in sepsis and critical illness have been inconsistent. Several trials have demonstrated benefits such as improved hemodynamic response, reduced ventilator time, and reduced mortality, while others report minimal to no significant impact (Table 3). The SCCM and an international task force led by the American College of Critical Care Medicine created guidelines in 2008 for corticosteroid treatment in sepsis, shock, acute respiratory distress syndrome (ARDS), and community-acquired pneumonia (CAP). These guidelines were subsequently updated by ESICM in 2017 [52]. The recommendations did not pertain to patients with chronic AI or address those with HIV or TB [52]. The task force supported the use of intravenous (IV) hydrocortisone at doses <400 mg/day for more than three days in patients with septic shock who are unresponsive to fluid resuscitation and vasopressor therapy. Conversely, there was moderate evidence against the use of corticosteroids in sepsis without shock. Corticosteroids were not recommended for the treatment of septic patients with trauma [52].

**Table 3:** Corticosteroids for severe infections or critical illness: a summary of practice guidelines and evidence.

Condition	Steroid Regimen	Outcomes and Measures	Comments/Knowledge Gaps
Acute Respiratory Distress Syndrome (ARDS) [79,80]	Dexamethasone 20 mg QD x 5 d, then 10 mg QD x 5 d	Decreased ICU mortality, hospital mortality, and all-cause mortality at day 60; Improved ventilator-free days at 28 days	For early ARDS, within 7 days of onset. Starting after 14 days likely harmful
Severe Community Acquired Pneumonia [35,81–83]	Hydrocortisone 200 mg total QD (or methylprednisolone 40-80 mg total QD) x 4-7 d, taper over 8-14 d	Decreased mortality, decreased incidence of endotracheal intubation and initiation of vasopressors by day 28	Severity defined by need for ICU-level respiratory support^*^ or Pulmonary Severity Index >130. Discrepancies between large RCTs, additional studies planned or ongoing
Septic Shock [77,78]	Hydrocortisone 200 mg total QD, with or without fludrocortisone 50 ug QD x 7 d	Decreased mortality, increased vasopressor-free days in some studies. No change in ventilator-free days	Mixed results from multiple large trials, may improve vasopressor-free days and mortality
Influenza [84–86]	N/a	N/a	Only retrospective studies suggesting harm. Most prospective studies have excluded influenza, or have small underpowered subgroups
SARS-CoV-2^*^[87]	Dexamethasone 6 mg QD x up to 10 d	Decreased mortality	Only in patients requiring supplemental oxygen. Suggestion of harm in patients not requiring oxygen
Pneumocystis pneumonia in HIV [88,89]	Prednisone 40 mg BID x 5 d, 40 mg QD x 5 d, 20 mg QD x 11 d	Decreased mortality, decreased need for mechanical ventilation	For moderate to severe PJP (hypoxemia based on PaO2 <70 mmHg on room air, or A-a gradient 35 mmHg or greater)

*Requiring mechanical ventilation with a positive end-expiratory pressure of at least 5 cm of water; high-flow nasal canula oxygen at FiO2 of 50% or more with a P/F <300; nonrebreather mask with P/F <300.

In 2024 the SCCM provided a focused update on the use of corticosteroids for the treatment of septic shock, ARDS, and CAP [90]. The update maintained the conditional recommendation for corticosteroid use in septic shock, including for patients requiring vasopressors. The most common regimens studied for septic shock were IV hydrocortisone at doses of 200–300 mg/day either in divided doses or as a continuous infusion for 5–7 days with or without tapering [90]. Previous studies used higher dose steroid regimens but were not recommended due to potential adverse effects including neuromuscular weakness, hypernatremia, and hyperglycemia, which varied significantly among studies [91]. The update highlighted that corticosteroids may have fewer benefits in the treatment of septic shock compared to ARDS; however, given the high global prevalence of septic shock, even small to moderate benefits from corticosteroid treatment may translate into substantial absolute benefits [90,92,93]. In a study of hospitalized patients from Uganda, all those with AI who received corticosteroids survived compared to none of those who did not receive corticosteroids [59]. Patients who died had higher serum cortisol levels. This finding suggests an exaggerated cortisol response and failure to clear cortisol from circulation due to tissue damage of septic shock. Accordingly, high cortisol levels may be a marker for severity of disease.

### Corticosteroid treatment in the setting of tuberculosis and HIV

The South African National Department of Health recommends the use of corticosteroids in the treatment of extrapulmonary TB, specifically in cases of TB meningitis and pericarditis. A high-dose steroid regimen is recommended for 2-4 weeks, followed by a gradual tapering depending on clinical progress [61]. A systematic review comprising nine trials with a total of 1,337 participants provided high-quality evidence supporting the use of adjuvant corticosteroids in the treatment of TB meningitis [94]. The review demonstrated that patients receiving corticosteroids (dexamethasone, methylprednisolone, or prednisolone) alongside anti-TB therapy had a nearly 22% reduction in mortality at follow-up periods of 3-18 months, with no significant increase in adverse effects [94,95]. A 2003 review of 11 randomized trials that included 1,814 participants who were treated with corticosteroids for pulmonary TB indicated a significant improvement in radiographic resolution of TB among those receiving steroid therapy [95,96]. However, while corticosteroids may confer benefits, especially in terms of early mortality reduction, the advantage in the context of rifampicin treatment regimens might be less pronounced due to increased metabolism of corticosteroids.

In PLWH, antiretroviral therapy can also alter corticosteroid pharmacokinetics. Ritonavir and cobicistat inhibit cytochrome P3A4 metabolism, potentially leading to increased corticosteroid concentrations and a heightened risk of adverse effects such as hyperglycemia and adrenal suppression. These interactions should be considered when using corticosteroids to treat sepsis in PLWH. Importantly, the trials reviewed indicated no adverse effects attributable to corticosteroid use [95]. Finally, in a meta-analysis from 2024 that included 7 randomized controlled trials comprising 1,410 PLWH and TB, the use of corticosteroids did not significantly impact all-cause mortality or the occurrence of serious adverse events [97]. Due to the high burden of HIV and TB in sub-Saharan Africa, corticosteroids for the treatment of critical illness may be particularly useful as subclinical AI from infectious causes may amplify CIRCI from sepsis [14,98].

## Discussion

Sub-Saharan Africa can be a challenging environment for sepsis management due to often limited human and material resources and the high burden of both HIV and TB, as well as other infections that can contribute to the immunopathophysiology of sepsis including AI. While autoimmune causes of AI predominate in other regions, infectious etiologies are more prevalent in sub-Saharan Africa. The overlap of symptoms associated with AI, HIV, and TB complicate the diagnosis of adrenal dysfunction in these patient populations, indicating a need for further exploration of AI in critically ill and septic patients. Subclinical AI from infectious causes may exacerbate CIRCI, thereby amplifying the potential benefits of corticosteroid therapy in septic patients in sub-Saharan Africa.

Sepsis is characterized by both hyperinflammatory and immunosuppressive responses, both of which can be detrimental. Clinical, transcriptomic, and biomarker signatures can be used to identify distinct sepsis subphenotypes and identify patients with the highest risk of mortality. Recent research from Uganda and Ghana suggests that PLWH and TB are more likely to demonstrate a hyperinflammatory, high-risk sepsis subphenotype. Despite the diversity of pathogens, the immune response patterns observed in Uganda and Ghana share similarities with those in HICs. This evidence highlights the need for clinical subphenotypes and point-of-care biomarkers that can identify high-risk patients, as well as those responsive to immunomodulation. For example, point-of-care sTNFR1 and Ang-2, combined with the clinical UVA mortality risk score, could perhaps assist in identifying high-risk and potentially corticosteroid-responsive sepsis subphenotypes.

We identified five studies from sub-Saharan Africa that assessed AI in PLWH between 2007 and 2018, encompassing 612 patients. The reported prevalence ranged from 5% to 35%. Similarly, eight studies from 2000 to 2020 that focused on TB revealed a prevalence of AI that ranged from 4% to 60%. The variability in these studies can largely be attributed to differences in definitions of AI, diagnostic methodologies, and variations in illness severity. This lack of consensus regarding the clinical definition of AI complicates the interpretation of these results including the prevalence of AI, which is a limitation of this review.

Although results of sepsis trials vary, current recommendations for adjuvant corticosteroid treatment in sepsis include moderate evidence for IV hydrocortisone for patients with septic shock who do not respond to fluid resuscitation or vasopressor therapy [52]. Importantly, the benefits observed in high-resource settings may not be applicable to sub-Saharan Africa. However, there is evidence supporting the benefit of adjunctive corticosteroid therapy for extrapulmonary and pulmonary TB. The South African National Department of Health’s endorsement of corticosteroids for managing extrapulmonary TB, particularly in TB meningitis and pericarditis, will be important to study to operationally determine the impact of corticosteroid therapy in addressing severe TB-related complications [61]. Given the numerous challenges in translating study results from high-income settings to low-resource environments, including Africa, our findings highlight the urgent need to investigate the optimal type and timing of corticosteroid treatment in different subphenotypes of patients with sepsis in sub-Saharan Africa. Due to the high prevalence of AI in patients with HIV and TB, which are also associated with high-risk sepsis clinical and immunological subphenotypes, there is a compelling rationale and equipoise for conducting such clinical trials [60,62].

This narrative review has limitations. First, the significant gap in clinical trials constrains our understanding of sepsis management in sub-Saharan Africa. Second, the reviewed studies had potential biases, including methodological differences and varied definitions of AI. These discrepancies contributed to heterogeneity of findings, notably the variable prevalence of AI among studies. Third, changes that have improved HIV and sepsis care in the past two decades may influence sepsis outcomes, yet this possibility is underexplored due to limited research in sub-Saharan Africa. Fourth, challenges in extrapolating results from high-income settings-based studies highlight the necessity for region-specific research to inform clinical practices. Given these limitations, optimal sepsis management in Africa remains unknown. Nonetheless, while awaiting clinical trial data from Africa, we propose that given the available evidence, that the use of corticosteroids should be considered in patients in sub-Saharan Africa with septic shock or sepsis and high mortality risk.

### Conclusion

The management of sepsis in sub-Saharan Africa requires a nuanced understanding of the unique immunological, epidemiological, and clinical landscape. Due to the high prevalence of AI in patients with HIV and TB, which are also associated with high-risk sepsis clinical and immunological subphenotypes, there is a compelling rationale and equipoise for conducting multi-center prospective cohort studies to better understand sepsis subphenotypes and ideally randomized controlled trials to assess corticosteroid therapy in the context of these subphenotypes. Future research is essential to validate these therapeutic approaches and establish evidence-based guidelines that consider the specific challenges faced in sub-Saharan Africa, which shoulders the highest burden of sepsis and associated mortality.

## Supporting information

S1 Table PubMed search strategy used to identify studies related to sepsis immuno-pathophysiology in sub-Saharan Africa. (DOCX)

S2 Table Comprehensive list of studies included in the narrative review of sepsis immunopathophysiology in sub-Saharan Africa. (DOCX)
