# Supplementary material for: A narrative review of the pathophysiology of sepsis in sub-Saharan Africa: Exploring the potential for corticosteroid therapy
Source: PLOS Glob Public Health. 2025 Apr 9;5(4):e0004429. doi: 10.1371/journal.pgph.0004429 (PMC11981229; doi:10.1371/journal.pgph.0004429)
Supplement: S1 Table — (DOCX) [file pgph.0004429.s001.docx]

| **Search Terms** | **Date of Search** | **Number of Results** |
| --- | --- | --- |
| ((((("Cytomegalovirus Infections"[Mesh] OR CMV)) OR ("Tuberculosis"[Mesh] OR "TB" OR "tuberculosis")) OR ("HIV"[Mesh] OR "human immunodeficiency virus" OR "HIV")) OR ("Acquired Immunodeficiency Syndrome"[Mesh] OR "AIDS")) AND (("Adrenal Insufficiency"[Mesh] OR "hypoadrenalism" OR "hypocortisolism" OR "Addison's Disease")) AND "Africa South of the Sahara"[Mesh] OR “SSA” OR “sub-saharan Africa” OR “Africa” | April 10, 2024 | 26 |

**Supplementary Table 1.** PubMed search strategy used to identify studies related to sepsis immuno-pathophysiology in sub-Saharan Africa
