# Supplementary material for: A narrative review of the pathophysiology of sepsis in sub-Saharan Africa: Exploring the potential for corticosteroid therapy
Source: PLOS Glob Public Health. 2025 Apr 9;5(4):e0004429. doi: 10.1371/journal.pgph.0004429 (PMC11981229; doi:10.1371/journal.pgph.0004429)
Supplement: S2 Table — (DOCX) [file pgph.0004429.s002.docx]

**Supplementary Table 2.** Comprehensive list of studies included in the narrative review of sepsis immunopathophysiology in sub-Saharan Africa.

| **Study** | **Country** | **Enrolled patients** | **Relevant findings** |
| --- | --- | --- | --- |
| Current sepsis management in sub-Saharan Africa | | | |
| **Dellinger (2004) [18]** | US | None – panel on sepsis guidelines | Developed guidelines on diagnosis and management of severe sepsis and septic shock. |
| **Evans (2021) [19]** | US | None – panel on sepsis guidelines | Evaluated and updated ninety-three guidelines for diagnosis and management of severe sepsis and septic shock. |
| **Rhodes (2016) [31]** | US | None – panel on sepsis guidelines | The Surviving Sepsis Guideline panel provided 93 statements on early management and resuscitation of patients with sepsis or septic shock. Overall, 32 were strong recommendations, 39 were weak recommendations, and 18 were best-practice statements. No recommendation was provided for four questions. |
| **Dünser (2012) [2]** | None – European Society of Intensive Care Medicine | None – literature review on sepsis management in resource-limited settings | Provides guidelines for acute and post-acute interventions for sepsis from the scarce evidence that exists for the management of pediatric and adult sepsis in resource-limited settings. The presented recommendations may help to improve sepsis management in middle- and low-income countries. |
| **Lal (2024) [20]** | Multiple countries | 2797 in- and outpatients who presented with fever | UVA score performed best in predicting mortality among febrile participants by the time follow-up compared with MEWS and qSOFA, across all four study sites. The UVA score could be a valuable tool for early identification, triage, and initial treatment guidance of high-risk patients in resource-limited clinical settings. |
| **Moore (2017) [21]** | Uganda | 5573 patients from six hospital-based cohort studies | The derived universal assessment (UVA) score for predicting mortality includes points for temperature, heart and respiratory rates, systolic blood pressure, oxygen saturation, Glasgow Coma Scale score and HIV serostatus. It outperforms MEWS and qSOFA. |
| **Schmedding (2019) [22]** | Gabon | 279 cases | UVA score performed best at predicting mortality compared to qSOFA, MEWS, and SIRS. An amalgamated qSOFA score applying the UVA thresholds for blood pressure and respiratory rate improved predictive ability in Gabon. |
| **Hazard (2022) [23]** | Rwanda | 573 hospitalized adult patients with acute infection | The admission UVA score and amalgamated qSOFA score had good predictive ability for mortality in adult patients admitted to hospital with acute infection in Rwanda compared to qSOFA and MEWS. |
| **Bonnewell (2021) [24]** | Tanzania | 597 adult patients hospitalized with febrile illness | NEWS and the UVA score performed favorably compared with MEWS, qSOFA, and SIRS in predicting in-hospital mortality among a hospitalized cohort of adults with febrile illness in northern Tanzania. |
| **Null (2024) [25]** | Uganda | 197 patients with severe sepsis | UVA score at 6 hours (adjusted OR [aOR] 1.26, 95%CI 1.10–1.45, p<0.001) was associated with in-hospital mortality. When adjusted for age and sex, improvement in the UVA risk group over 6 hours was associated with a non-statistically significant 43% decrease in odds of mortality. |
| **Burke (2023) [26]** | UK and Malawi | None – literature review on HIV patients in low- and middle-income countries | Ten studies published since 2003 investigated interventions that aimed to reduce mortality in hospitalised adults with HIV and weren’t restricted to people with a defined disease diagnosis. Inpatient trials of diagnostics, therapeutics or a package of interventions to reduce mortality should be a research priority. |
| **Peter (2016) [27]** | South Africa, Tanzania, Zambia, and Zimbabwe | 8728 patients with HIV in ten hospitals across four African countries | Bedside LAM-guided initiation of anti-tuberculosis treatment in HIV-positive hospital inpatients with suspected tuberculosis was associated with reduced 8-week mortality. The implementation of LAM testing is likely to offer the greatest benefit in hospitals where diagnostic resources are most scarce and where patients present with severe illness, advanced immunosuppression, and an inability to self-expectorate sputum. |
| **Gupta-Wright (2018) [28]** | Malawi and South Africa | 2600 HIV-positive adults across two hospitals | Urine-based tuberculosis screening did not reduce overall mortality in all HIV-positive inpatients, but might benefit some high-risk subgroups. Implementation could contribute towards global targets to reduce tuberculosis mortality. |
| **Andrews (2017) [29]** | Zambia | 212 adults with sepsis and hypotension in the ED | Among adults with sepsis and hypotension, most of whom were positive for HIV, in a resource-limited setting, a protocol for early resuscitation with administration of intravenous fluids and vasopressors increased in-hospital mortality compared with usual care. |
| **Maitland (2011) [30]** | Uganda, Kenya, and Tanzania | 3141 children with severe febrile illness and impaired perfusion | Fluid boluses significantly increased 48-hour mortality in critically ill children with impaired perfusion in these resource-limited settings in Africa. |
| **Mer (2019) [32]** | None – textbook chapter | None – textbook chapter | This chapter outlines important elements for the general supportive care for patients with sepsis in resource-limited settings. It recommends low-dose corticosteroids in septic patients with refractory shock. |
| **The immune response to sepsis** | | | |
| **Hotchkiss (2003) [33]** | US | None – literature review on sepsis | The article generally examines the evolving concepts of sepsis and discusses new and potential therapies. |
| **Torres (2015) [34]** | Spain | 112 patients from 3 Spanish teaching hospitals | Patients with severe community-acquired pneumonia and a high initial inflammatory response who received methylprednisolone experienced less treatment failure than those who didn’t receive methylprednisolone. |
| **Cajander (2024) [5]** | Multiple European countries | None – literature review | The review provides an overview of the current state of immune profiling in sepsis, including its use, current challenges, opportunities for progress. |
| **Dequin (2023) [35]** | France | 800 patients with severe community-acquired pneumonia | Patients with severe community-acquired pneumonia in ICU who received hydrocortisone had a lower risk of death by day 28 than those who received placebo. |
| **Phenotypes of the immune response to sepsis** | | | |
| **Hack (1989) [36]** | Netherlands | 37 patients with sepsis in an Amsterdam MICU | IL-6 on admission appeared to be of prognostic significance: levels were higher in septic patients who subsequently died than in those who survived [P = .0003), in particular when only patients with septic shock were considered {P < .0001). |
| **Hack (1992) [37]** | Netherlands | 47 patients in the ICU with sepsis | Patients who died had higher IL-8 levels on admission than the patients who survived. Higher IL-8 levels were associated with higher lactate, IL-6, elastase-a1-antitrypsin, and C3a levels and inversely associated with leukocyte and platelet levels. |
| **Marty (1994) [38]** | UK | None – cell culture experiment | Under conditions mimicking sepsis, melatonin and other structurally related indoleamine compounds have effects on NFκB activation and cytokine expression, GSH, mitochondrial membrane potential, and metabolic activity in endothelial cells. |
| **Song (2019) [39]** | Korea | 142 patients in the ED at a Korean university | Serum IL-6 levels could discriminate sepsis from controls and could distinguish septic shock from sepsis. Twenty-eight-day mortality was significantly higher in the group with high IL-6 (≥ 348.92 pg/mL) than in the group with low IL-6 (< 348.92 pg/mL) (P = 0.008). |
| **Anderson (2019) [40]** | US | 400 patients with sepsis | An admission sTNFR1 concentration > 8861 pg/ml identified patients with increased mortality in both the derivation (RD 21.6%) and validation (RD 17.8%) populations. Among immunocompetent patients, an IL8 concentration > 94 pg/ml identified patients with increased mortality in both the derivation (RD 17.7%) and validation (RD 27.0%) populations. |
| **Fumeaux (2002) [41]** | Switzerland | 98 patients | HLA-DR molecules are re-endocytosed and retained intracellularly in monocytes from patients with septic shock, and that this phenomenon is partially mediated by IL-10. IL-10 may represent a future target for immunomodulating patients with the sepsis syndrome or critically ill patients at risk of developing infections. |
| **Marie (1996) [42]** | France | 26 patients with sepsis | The systemic inflammatory response syndrome is associated not only with the exacerbation of the production of proinflammatory cytokines but also with the increased release of many anti-inflammatory actors, including specific interleukin-1 and tumor necrosis factor inhibitors and such cytokines as interleukin-10 and TGF-β 1. |
| **Davenport (2016) [43]** | UK | 265 adult patients in UK ICUs with sepsis due to community-acquired pneumonia and evidence of organ dysfunction | The presence of SRS1 (detected in 108 [41%] patients in discovery cohort) identifies individuals with an immunosuppressed phenotype that included features of endotoxin tolerance, T-cell exhaustion, and downregulation of human leucocyte antigen (HLA) class II. SRS1 was associated with higher 14 day mortality than was SRS2 (discovery cohort hazard ratio (HR) 2·4, 95% CI 1·3–4·5, p=0·005; validation cohort HR 2·8, 95% CI 1·5–5·1, p=0·0007). |
| **Seymour (2019) [44]** | US | 63858 patients with sepsis | 4 clinical phenotypes were identified that correlated with host-response patterns and clinical outcomes, and simulations suggested these phenotypes may help in understanding heterogeneity of treatment effects. |
| **Phenotypes of the immune response to sepsis in sub-Saharan Africa** | | | |
| **Cummings (2022) [45]** | US | None - review | Leveraging advances in high-dimensional host response profiling and computational methods, identification of patient subgroups defined by distinct clinico-molecular features, and pathobiological mechanisms (“endotypes”) represents a promising approach to achieve biological deconvolution of the heterogenous sepsis phenotype. |
| **Cummings (2024) [46]** | Uganda | 43 patients | Patients who died showed heterogeneous expansion of polymorphonuclear myeloid-derived suppressor cells, with increased and decreased abundance of CD16^−^PD-L1^dim^ and CD16^bright^PD-L1^bright^ subsets, respectively, significantly associated with mortality. |
| **Cummings (2023) [47]** | Uganda | 260 adults with suspected sepsis | Among 14 biomarkers, soluble tumor necrosis factor receptor 1 (sTNFR1) and angiopoietin 2 (Ang-2) demonstrated the greatest importance for mortality prediction in machine learning models. A clinicomolecular model integrating sTNFR1 and Ang-2 with the Universal Vital Assessment (UVA) risk score optimized 30-day mortality prediction across multiple performance metrics. |
| **Cummings (1999) [48]** | Uganda | 157 hospitalized patients living with HIV | The study suggests a pathobiological relationships between LAM, TB dissemination, innate cell activation, and evasion of host immunity in severe HIV/TB. |
| **Cummings (2024) [49]** | Uganda | 128 hospitalized patients with suspected sepsis | Patients assigned to SRS-1 were predominantly (80.0% [24/30]) persons living with HIV with advanced immunosuppression and frequent tuberculosis. Mortality at 30-days differed significantly by endotype and was highest (48.1%) in SRS-1. |
| **Chenoweth (2024) [50]** | Ghana | 120 hospitalized patients with sepsis | Data dimensional reduction reveals dynamic gene expression patterns that describe cell type-specific molecular phenotypes including a dysregulated myeloid compartment shared between sepsis and COVID-19. The gene expression signatures reported here define a landscape of host response to sepsis that supports interventions via targeting immunophenotypes to improve outcomes. |
| Pathophysiology of adrenal insufficiency and critical illness-related corticosteroid insufficiency (CIRCI) | | | |
| **Mofokeng (2022) [13]** | UAE and South Africa | None – literature review | In Africa, the diagnostic criteria for PAI do not universally accord with conventional criteria, and there is a heavy reliance on clinical suspicion and biochemistry, including random cortisol of <400 nmol/L, rather than the tetracosactide test where stimulated cortisol <500–550 nmol/L confirms the diagnosis. |
| **Atiase (2024) [51]** | Ghana | None – literature review | Adrenal insufficiency refers to inadequate production of glucocorticoids, mineralocorticoids, or both by the adrenal glands. Glucocorticoid replacement is the mainstay of management, and this may be combined with mineralocorticoids in the case of primary adrenal insufficiency. |
| **Annane (2017) [12]** | Multiple Countries | None – literature review | Three major pathophysiologic events were considered to constitute CIRCI: dysregulation of the hypothalamic–pituitary–adrenal (HPA) axis, altered cortisol metabolism, and tissue resistance to glucocorticoids. |
| **Annane (2017) [52]** | Multiple Countries | None – CIRCI diagnosis and management guidelines | An update of the 2008 CIRCI guidelines and evidence-based recommendations for the use of corticosteroids in critically ill patients with sepsis and septic shock, acute respiratory distress syndrome, and major trauma have been developed by a multispecialty task force. |
| Prevalence of adrenal insufficiency in patients living with HIV in sub-Saharan Africa | | | |
| **Akase (2019) [53]** | Nigeria | 350 adult patients living with HIV | The biochemical evidence of hypocortisolism was common among patients infected with HIV, associated with a longer duration of HIV infection. However, none of CD4 counts, clinical features or HAART regimen were associated with hypocortisolism. |
| **Kibirige (2014) [54]** | Uganda | None – literature review | Adrenal insufficiency, diabetes mellitus and calcium-vitamin D abnormalities were the most prevalent and frequently reported endocrine disorders among adult patients with tuberculosis in Africa. A meticulous endocrine evaluation among tuberculosis patients with suspected endocrine abnormalities should be encouraged in Africa and other high TB endemic regions. |
| **Odeniyi (2017) [55]** | Nigeria | 100 patients who had not undergone HIV treatment | AI, at subclinical level, was less frequent in those with PTB and HIV co-infection. |
| **Odeniyi (2013) [56]** | Nigeria | 113 patients who had not undergone HIV treatment | Adrenocortical insufficiency is common in persons with HIV infection, occurring in about 34.8% of patients studied. Clinically evident adrenocortical insufficiency is uncommon in persons with HIV. |
| **Akase (2018) [57]** | Nigeria | 350 patients living with HIV | There is a high prevalence of hypocortisolism among HIV patients by biochemical testing, especially those who have been on ARVs for a longer duration. Hypocortisolism cannot be predicted based on the participants' WHO clinical stage of disease, CD4 cell count, or the treatment regimen. |
| **Ekpebegh (2011) [58]** | South Africa | 66 hospitalized patients living with HIV | The prevalence of hypoadrenalism was 27% with a high occurrence of contributory factors (cytomegalovirus infection in 100% and tuberculosis in 68.2% of the study subjects). Typical features of hypoadrenalism included: hyponatremia, 19.6%; hyperkalemia, 6.1%; mucosal hyperpigmentation, 12%, and loss of axillary hair, 15%. |
| **Meya (2007) [59]** | Uganda | 113 hospitalized patients with HIV | The major admitting diagnosis among both groups was tuberculosis (38% in patients with AI and 41% in those without). Risk factors for AI included rifampicin, septrin, tachycardia, HIV stage IV disease, eosinophilia, and hyponatremia. Additional major admitting diagnoses of the AI patients included Kaposi sarcoma, cryptococcal meningitis, bronchopneumonia, and *Pneumocystis jiroveci*. |
| **Kibirige (2024) [60]** | Uganda | 4044 patients across 46 studies | The pooled prevalence of AI was 33% (95% CI, 22%–45%; I2 = 97.7%, P < .001) in participants with tuberculosis and 28% (95% CI, 18%–38%; I2 = 98.9%, P < .001) in those with HIV. |
| Prevalence of adrenal insufficiency in patients with tuberculosis in sub-Saharan Africa | | | |
| **(2014) [61]** | South Africa | None – Tuberculosis guidelines | The guidelines go through tuberculosis transmission, diagnosis, registration, treatment, interaction with HIV, and multi-drug resistance. |
| **Azeez (2021) [62]** | Nigeria and Sierra Leone | 809 patients across 8 studies | The frequency of adrenal insufficiency in patients with pulmonary TB can be as high as 50%. The presence of low blood pressure, low blood glucose, multidrug-resistant TB, and generalized skin hyperpigmentation is a pointer to the possibility of adrenal insufficiency in these patients. |
| **Naggirinya (2020) [63]** | Uganda | 272 patients with tuberculosis | 117 (43%) had drug-resistant TB. Mean cortisol levels were lower in participants with drug-resistant than susceptible TB (p < 0.001). In multivariable analyses, drug-resistant TB (p < 0.001), treatment duration > 1 month (p = 0.002) and abdominal pain (p = 0.038) were significantly associated with FAI. Early morning serum cortisol levels should be quantified in TB-HIV co-infected patients with drug-resistant TB. |
| **Mabuza (2020) [64]** | South Africa | 75 patients with tuberculosis | 28 (37.3%) were classified as Adrenal Insufficient AI. A low fasting serum glucose, a positive GeneXpect, a low CD4 count with a minimum of ten signs and symptoms constitute a discriminator for AI in TB-suspect patients, necessitating treatment initiation to save patient lives in laboratory resource-limited settings. |
| **Broodryk (2010) [65]** | South Africa | 73 hospitalized patients with tuberculosis | Five patients (6.85%) had a blunted response to the ACTH stimulation test which identifies some form of adrenal insufficiency. None of the patients had an increased ACTH concentration. This finding excludes PAI and the normal ACTH concentrations in these 5 patients are highly suggestive of secondary-or tertiary adrenal insufficiency. |
| **Beadsworth (2008) [66]** | Malawi | 51 patients with tuberculosis | Of 43 patients HIV-tested, 38 (88.3%) were HIV-positive and 15.7% died within the first month. At 3 months, 11 (21.6%) were known to have died. Adequate cortisol levels were found in 49/51 (95.9%) before commencing RMP. Neither of the two with reduced response died. All 34 patients revealed adequate cortisol responses at 2 weeks. |
| **Francois (2006) [67]** | South Africa | 28 hospitalized patients with tuberculosis | Rifampicin did not additionally impair adrenocortical function during the initial period of therapy. The high cortisol/DHEA-S ratio might be of clinical relevance. |
| **Odeniyi (2011) [68]** | Nigeria | 44 patients with tuberculosis | Adrenocortical insufficiency, mostly at the subclinical level, is common in persons with PTB infection, occurring in about 23% of patients. We therefore recommend that basal cortisol levels should not be used to detect adrenocortical insufficiency; rather stimulation tests should be used to exclude or confirm suspected adrenocortical insufficiency in patients with PTB. |
| **Namulema (2009) [69]** | Uganda | 200 patients with tuberculosis | 1/8 patients had AI, which demonstrates that adrenal insufficiency is a common complication of PTB in a Ugandan setting. The factors associated with adrenal insufficiency in this study were, abdominal pain, salt craving, muscle pains and absence of nausea. |
| **Kaplan (2000) [70]** | South Africa | 40 patients with tuberculosis | Primary hypoadrenalism, as assessed by the 1 microg ACTH test, was uncommon in a cohort of ill, hospitalized patients with active PTB, irrespective of HIV status. |
| Role of corticosteroids in sepsis management | | | |
| **D’Emmanuele (2003) [71]** | Italy | None -laboratory experiment with rats | The beneficial effect of dexamethasone in endotoxemia could be ascribed, at least in part, to its ability to interfere with KATP channel activation induced by LPS. This interaction may explain the improvement of vascular reactivity to PE, mediated by DEX, in LPS-treated rats, highlighting a new pharmacological activity to the well-known anti-inflammatory properties of glucocorticoids. |
| **Heming (2018) [72]** | France | Unknown – literature review on two clinical trials with an unlisted number of participants | The combination of hydrocortisone and fludrocortisone significantly reduces mortality in septic shock. Recently, potential immune effects of mineralocorticoids via non-renal mineralocorticoid receptors have gained popularity. |
| **Prigent (2004) [73]** | France | None – literature review | This review describes current knowledge on the mechanisms that underlie glucocorticoid insufficiency in sepsis and the molecular action of glucocorticoids. |
| **Shankar-Hari (2024) [4]** | Multiple countries | Review | Position paper to reframe the dysregulated host immune responses in sepsis as altered homoeostasis with pathological disruption of immune-driven resistance, disease tolerance, resilience, and resolution mechanisms |
| **Annane (2002) [74]** | France | 300 patients with septic shock | A 7-day treatment with low doses of hydrocortisone and fludrocortisone significantly reduced the risk of death in patients with septic shock and relative adrenal insufficiency without increasing adverse events. |
| **Sprung (2008) [75]** | Multiple countries | 499 patients with sepsis across 52 ICUs | At 28 days, there was no significant difference in mortality between patients in the two study groups who did not have a response to corticotropin (39.2% in the hydrocortisone group and 36.1% in the placebo group, P=0.69) or between those who had a response to corticotropin (28.8% in the hydrocortisone group and 28.7% in the placebo group, P=1.00). |
| **Keh (2016) [76]** | Germany | 380 adult patients across 24 ICUs with severe sepsis without septic shock | Among adults with severe sepsis not in septic shock, use of hydrocortisone compared with placebo did not reduce the risk of septic shock within 14 days. These findings do not support the use of hydrocortisone in these patients. |
| **Venkatesh (2018) [77]** | Multiple countries | 3800 patients with septic shock | Among patients with septic shock undergoing mechanical ventilation, a continuous infusion of hydrocortisone did not result in lower 90-day mortality than placebo. |
| **Annane (2018) [78]** | France | 1241 patients in the ICU with septic shock | 90-day all-cause mortality was lower among those who received hydrocortisone plus fludrocortisone than among those who received placebo. |
| **Chaudhuri (2024) [79]** | Multiple countries | None –panel discussing corticosteroid use for sepsis | The panel provided a conditional recommendation to administer corticosteroids for patients with septic shock and critically ill patients with ARDS and a strong recommendation for use in hospitalized patients with severe CAP. The panel also recommended against high dose/short duration administration of corticosteroids for septic shock. The panel was unable to provide specific recommendations addressing corticosteroid molecule, dose, and duration of therapy. |
| **Mirza (2021) [80]** | US | None – literature review on use of corticosteroids in sepsis | Ten articles were reviewed. Among those, two demonstrated improved clinical outcomes, two showed both improved clinical outcomes and decreased mortality, three showed increased adverse eﬀects, and the remaining one showed unequivocal results. |
| **Yerke (2020) [81]** | US | None – literature review | Hydrocortisone therapy is likely to demonstrate maximal benefit when initiated on patients with septic shock and organ failure refractory to vasopressor therapy and should be used judiciously in other settings as it comes without a demonstrated benefit in mortality and increased potential for adverse effects. |
| **Fang (2019) [82]** | China and US | 9564 patients with sepsis across 37 clinical trials | Corticosteroids were associated with reduced 28-day mortality, increased shock reversal at day 7, and vasopressor-free days, with decreased intensive care unit length of stay, the Sequential Organ Failure Assessment score at day 7, and time to resolution of shock. |
| **Corticosteroid treatment in the setting of tuberculosis and HIV** | | | |
| **Prasad (2016) [83]** | India and UK | 1337 patients with Tuberculosis across 9 trials | Corticosteroids reduce mortality from tuberculous meningitis, at least in the short term. Corticosteroids may have no effect on the number of people who survive tuberculous meningitis with disabling neurological deficit, but this outcome is less common than death, and the CI for the relative effect includes possible harm. |
| **Siddiqui (2010) [84]** | India | Review | Corticosteroids significantly decrease the risk of pleural thickening in patients with tubercular pleural effusion; the clinical significance of this finding, however, is unclear. Recently, it has been demonstrated that use of corticosteroids improve the morbidity in HIV co-infected patients with paradoxical TB immune reconstitution inflammatory syndrome (IRIS). However, evidence favouring the use of corticosteroids in other clinical situations is sparse or lacking. Likewise, the biological mechanisms underlying their beneficial effect in TB meningitis and pericarditis remain poorly understood. |
| **Smego (2003) [85]** | Pakistan | 1814 patients with tuberculosis across 11 trials | The adjunctive use of systemic corticosteroid therapy can safely provide significant early and prolonged clinical and radiographic benefits in selected patients with advanced pulmonary tuberculosis. |
| **Pu (2024) [86]** | China | 1410 patients living with HIV across 7 studies | Corticosteroid use did not significantly reduce all-cause mortality (RR = 0.91, 95% CI: .79–1.04, P = .17) and did not significantly increase serious adverse events (RR = 0.96, 95% CI: .82–1.13, P = .63). |
| **(2024) [14]** | United Nations | None – global AIDS report | The report breaks down the current state of the HIV pandemic, discusses HIV services and solutions, progress on UN goals, and regional reports and breakdowns. |
| **(2023) [87]** | United Nations | None – global tuberculosis report | The report discusses the Tuberculosis global burden, diagnosis, treatment, prevention, financing, and research. |
